# Assessing the feasibility of a clinical trial to evaluate an advanced practice physiotherapy model of care in chronic pain management: a feasibility study

**DOI:** 10.1186/s40814-023-01352-9

**Published:** 2023-07-17

**Authors:** Jordan Miller, Tom Doulas, Etienne J. Bisson, Abey Abebe, Mulugeta Chala, Chad McClintock, Kevin Varette, Kyle Vader, François Desmeules, Kadija Perreault, Catherine Donnelly, Randy Booth, Andrews K. Tawiah, Scott Duggan

**Affiliations:** 1 School of Rehabilitation Therapy, Queen’s University, Louise D Acton Building, 31 George Street, Kingston, ON K7L 3N6 Canada; 2 Health Services and Policy Research Institute, Faculty of Health Sciences, Queen’s University, 18 Barrie Street, Kingston, ON K7L 3N6 Canada; 3 Chronic Pain Clinic, Kingston Health Sciences Centre — Hotel Dieu Hospital site, Jeanne Mance 3, 166 Brock Street, Kingston, ON K7L 5G2 Canada; 4 Department of Anesthesiology and Perioperative Medicine, Kingston Health Sciences Center, Queen’s University, Victory 2, 76 Stuart Street, Kingston, ON K7L 2V7 Canada; 5 Centre for Neuroscience Studies, Queen’s University, Botterell Hall, 18 Stuart Street, Kingston, ON K7L 3N6 Canada; 6 School of Rehabilitation, Université de Montréal, 7077 Park Avenue, Montréal, Québec H1T 2M4 Canada; 7 Centre Interdisciplinaire de Recherche en Réadaptation Et Intégration Sociale, Centre de Santé Et de Services Sociaux de La Capitale-Nationale, 525 Wilfrid-Hamel, Québec City, Québec G1M 2S8 Canada; 8 Department of Rehabilitation, Université Laval, Pavillon Ferdinand-Vandry, Suite 4247, Québec City, Québec G1V 0A6 Canada; 9 Faculty of Rehabilitation Medicine, University of Alberta, Edmonton, AB T6G 2G4 Canada

**Keywords:** Chronic pain, Advanced practice physiotherapy, Feasibility study, Interprofessional

## Abstract

**Background:**

Chronic pain management is challenging for health systems worldwide. Clinical practice guidelines recommend interprofessional chronic pain management, but chronic pain clinics often have lengthy wait-lists. Advanced practice physiotherapists (APP) in orthopedic clinics and emergency departments have provided effective care and reduced wait times. The purpose of this study is to determine the feasibility of a clinical trial to evaluate the effects of integrating an APP into a chronic pain clinic setting. The primary objectives are as follows: (1) determine the feasibility of implementing trial methods by evaluating participant recruitment rates, retention, and assessment completion; (2) determine the feasibility of implementing the APP model of care by monitoring care provided and treatment fidelity; and (3) assess contextual factors that may influence implementation of the APP model of care by exploring the perspectives of patient participants and healthcare providers related to the model of care.

**Methods:**

This will be a single-arm feasibility study with embedded qualitative interviews to assess contextual factors influencing implementation by exploring participant and provider perspectives. Approximately 40 adults with chronic musculoskeletal pain referred for care at an interprofessional chronic pain clinic will be invited to participate in the feasibility study. Approximately 10–12 patient participants and 5–10 health professionals from the interprofessional team will be interviewed using an interpretive description approach. The APP model of care will involve participants seeing a physiotherapist as the first point of contact within the interprofessional team. The APP will complete an initial assessment and make care recommendations. Outcome measures planned for the full trial will be reported descriptively, including pain severity, pain interference, health-related quality of life, psychosocial risk factors for chronic pain, treatment satisfaction, perceived change, healthcare utilization, and healthcare costs over one year.

**Discussion:**

This study will inform plans to implement a full-scale study to evaluate the impact of an APP model of care in an interprofessional chronic pain management program. The results of the full study are intended to inform stakeholders considering this model to improve patient-centered and health system outcomes in interprofessional pain management program settings.

**Trial registration:**

ClinicalTrials.gov, NCT05336903 (Registered April 5, 2022).

**Supplementary Information:**

The online version contains supplementary material available at 10.1186/s40814-023-01352-9.

## Background

Chronic pain is a leading contributor to years lived with disability worldwide [1]. Direct and indirect costs associated with chronic pain are estimated at US $38.3 to US $40.4 billion per year in Canada, with musculoskeletal pain representing the majority of the costs [2]. Clinical practice guidelines recommend interprofessional chronic pain management based on high-quality evidence of effectiveness and cost-effectiveness [3–5]; however, access to interprofessional chronic pain management programs is a challenge for many individuals, with median wait times across Canada of over months duration [6–8]. This is in contrast to the quality standards for chronic pain management in Ontario, Canada, which indicate that people with chronic pain should receive an appointment with a chronic pain program within three months of referral [9].

Among individuals on waitlists for interprofessional chronic pain clinics, Choinière et al. found that almost two-thirds reported severe pain (≥ 7/10) that interferes with activities of daily living [10]. In addition, they found a high prevalence of both depression (50% of patients) and suicidal ideation (35% of patients) among individuals on waitlists for interprofessional chronic pain clinics. Waiting for chronic pain services is also costly to the health system, with reported median healthcare costs of US $1462 per month for those on waiting lists [11]. Given the personal suffering and healthcare costs incurred as individuals await chronic pain services, strategies to reduce waitlists and increase cost effectiveness are urgently needed to improve individual and societal outcomes of chronic pain.

One strategy that may contribute to the provision of guideline adherent care, while reducing waitlists, off-loading physician and nurse practitioner workload, and improving cost-effectiveness for people living with chronic pain, is incorporating an advanced practice physiotherapist (APP) into the interprofessional team. APP models of care involve physiotherapists (PTs) working to their full scope of practice using advanced knowledge, clinical reasoning skills, and experiences to help manage patients with complex conditions in an interprofessional team setting [12–16]. APP roles have been implemented in orthopedic care and emergency department settings, among other settings. For example, in an outpatient orthopedic clinic setting, APPs are commonly the first point of contact, triage patients appropriate for surgery, and refer to the surgeon when needed [14]. Research suggests that APP models of care in these settings can improve access and quality of care for patients with musculoskeletal conditions while maintaining safety and high levels of patient satisfaction [13–15, 17–19].

Musculoskeletal pain is the most prevalent classification of chronic pain [2] and among the most common reasons for seeking specialized health care for pain [20–22]. PTs have the competencies to complete a comprehensive assessment to identify physical, psychological, and social factors contributing to pain and disability among people with musculoskeletal pain and have successfully taken on advanced practice roles in other settings with musculoskeletal pain conditions [13]. Additionally, they have competencies in collaborative interprofessional care that will allow them to identify and engage the most appropriate team members in the care of people with chronic pain [23]. PTs may be well-suited to take on this first-contact role within the team for the subgroup of people with musculoskeletal pain seeking interprofessional pain care [1, 2].

APP models have yet to be implemented and evaluated in interprofessional chronic pain clinic settings; however, evidence from other healthcare settings suggests that this model of care has the potential to improve access to care while reducing costs. A review by Vedanayagam et al. [24] suggested that APP models of care can lead to improved access to care while maintaining a high degree of diagnostic accuracy, effective management, and similar patient health outcomes to usual care models across multiple settings. Another review of economic evaluations of APP models of care found that APP integration may result in reduced health system costs [25]. The potential mechanisms for cost reduction include the following: a reduction in referrals for surgery, APP salaries are less than physician salaries, and an attenuation of medications prescribed.

In order to successfully implement a full-scale trial to evaluate the effects of APPs working within chronic pain clinics, there are unique aspects of an interprofessional chronic pain clinic setting and patient population that need to be considered. There is the fundamental need to provide a comprehensive assessment and incorporate patient care within a wider interprofessional team with professionals with diverse training backgrounds and perspectives (e.g., social workers, psychologists, occupational therapists, nurse practitioners, anesthesiologists). Individuals with chronic pain often present with complex medical histories and higher rates of psychological and social factors related to pain that are likely to benefit from an interprofessional approach to care [26–28]. Integrating expertise from various disciplines is considered an effective approach to improve patient care for complex health conditions [29–31], and there is evidence that PTs can provide effective collaborative care [32–35].

Interventions with multiple components and care providers, such as a new APP model of care, introduce elements of complexity to the successful implementation of a full-scale clinical trial in an interprofessional chronic pain clinic setting. Some of these challenges include unique staffing arrangements, delivery of interventions in a dynamic setting, communication between healthcare providers (HCPs), recruitment of participants with competing priorities, and uncertainty around participant retention [36–38]. Medication discussion and management are often part of a comprehensive assessment and necessary treatment plan for individuals with chronic pain [39–41]. PTs in Canada are currently not able to prescribe medications, without medical directives, which may mean the APP model of care requires frequent involvement of physicians within the pain clinic, reducing the potential of the model to reduce physician workload.

Another rationale for this feasibility study is the need to understand contextual factors that may influence implementation within a fully powered trial. Several qualitative studies have been conducted related to the perceived barriers and facilitators of an APP model of care in other healthcare settings [42–46], but it is unknown how this model of care will be received by the patient participants and HCPs in a chronic pain clinic setting in the Canadian health system context. Themes related to APP knowledge, experience, and skills have been cited as drivers to the successful implementation of this model. A qualitative study in the Dutch healthcare system suggested that barriers related to a misalignment of physician values, a reluctancy to transfer care to APPs, and a lack of clarity of the value added from an APP model present significant challenges to the implementation of this model of care [47]. Being aware of these factors, as they relate to this feasibility study, will help inform a future full trial; perceived satisfaction and value from the model of care are vital to its uptake in a real-world setting.

Feasibility studies are often considered an essential prerequisite to large-scale studies that require a great deal of time, money, and resources [48]. The feasibility of an APP model of care needs to be tested in the chronic pain clinic setting to understand the perspectives of participants and their providers towards this model of care prior to conducting a fully powered clinical trial. The future aim of this line of research is to assess the impact of integrating an APP as the first point of contact within interprofessional chronic pain clinics. The primary objectives in a full trial will focus on participant health outcomes, care provided to participants, chronic pain clinic flow, the alignment of care with quality standards [9], and cost utility of the APP model in comparison to the usual physician- or nurse practitioner-led care. These objectives will be addressed in a full-scale clinical trial to be conducted following evidence of feasibility.

The aim of the current study is to evaluate the feasibility of conducting a future trial to evaluate the effects of implementing an APP role within a chronic pain clinic setting. The primary objectives of the feasibility study are as follows:

1. To determine the feasibility of implementing the trial methods by evaluating participant recruitment rates, retention, and assessment completion over a 12-month period
2. To determine the feasibility of implementing the new APP model of care by monitoring the care provided and treatment fidelity
3. To assess the contextual factors that may influence implementation of an APP-integrated model of care by exploring the perspectives of patient participants and healthcare providers.

## Methods

### Study design

This is a single-arm study where the focus is on testing the feasibility of the proposed full trial procedures [49]. Single-arm designs are considered appropriate where the goal of the study is to collect preliminary information regarding the feasibility and safety of a trial [50]. A feasibility, rather than a pilot, design is a pragmatic choice for the current study since the comparison arm, the usual physician- or nurse practitioner-led model, is already being used. The electronic surveys involved in the study can easily be integrated into a full trial across multiple clinical sites. Medical Research Council (MRC) guidelines for the development and evaluation of complex interventions have been used to guide this feasibility study [36, 37]. This study will collect data from participants with chronic pain and their providers to assess the feasibility of implementing and evaluating an APP role in an interprofessional chronic pain clinic setting. Both quantitative and qualitative data will be collected to assess the feasibility of the design (recruitment, data collection, retention), feasibility of intervention implementation (care provided, fidelity), and contextual factors that may influence implementation of the model of care (acceptability; barriers, facilitators, and strategies for refining the implementation; impact on the clinical process).

A SPIRIT checklist [51], modified for a feasibility study, has been completed and provided in Additional file 1.

### Setting

The Chronic Pain Clinic at Kingston Health Sciences Centre (KHSC) is located in Kingston, Ontario, Canada. The clinic is an interprofessional program that provides chronic pain management services to adult populations. The team includes five anesthesiologists, one neurosurgeon, four independently practicing nurse practitioners, three registered nurses, two PTs, two occupational therapists, one psychologist, and two social workers. The team is further supported by administrative staff and a research coordinator.

### Recruitment and consent

We will invite consecutive adult patient participants (18 years and older) with chronic musculoskeletal pain (> 3 months) referred to the KHSC Chronic Pain Clinic until we recruit approximately 40 patient participants. Forty participants were determined sufficient to address our feasibility outcomes using a practicality approach [51] and is within an acceptable rule of thumb range for pilot trials [52]. Potential participants will be triaged by a registered health professional from the Chronic Pain Clinic interprofessional team based on referral information and study inclusion/exclusion criteria. A registered practical nurse (RPN) will ask potential participants for consent to be contacted by a research team member. A research associate will then contact the potential participants to provide full information about the trial and invite them to participate. Consenting participants will then be enrolled in the study prior to completing the baseline assessment and scheduling an appointment with the APP.

### Inclusion and exclusion criteria

Inclusion criteria are as follows:

1. Chronic musculoskeletal pain (pain > 3 months in duration) (e.g., neck pain, thoracic pain, limb pain, low back pain) -OR-
2. Chronic widespread pain (e.g., fibromyalgia, myofascial syndrome) -AND-
3. Eighteen years of age or older -AND-
4. Must be able to read, write, and speak English.

Exclusion criteria as identified on referral package are as follows:

1. Primary reason for referral stated as medication change or interventional pain management (e.g., injections, nerve block).
2. Primary reason for referral is stated as headache or migraine.
3. Reason for referral includes chronic pelvic pain.
4. Referral indicates the individual has an untreated addiction, mental health disorder, or substance use disorder.
5. Referral indicates cancer-related pain.
6. Referral indicates medical “red flags” suggestive of non-musculoskeletal etiology of symptoms (e.g., unexplained symptoms, sudden weight loss, urinary retention, saddle anesthesia, evidence of upper motor neuron lesion, or fever).
7. Referral states visceral pain or abdominal pain.

### Blinding and allocation concealment

Participants will be invited to participate in the study with an understanding that they will be assigned to either a model of care in which they see a physician or nurse practitioner first or a model of care in which they see an APP first, even though this is a single-arm feasibility study, and all participants will be assigned to the APP model of care. This partial masking at the time of consent and initial assessment was determined to be important to provide accurate estimates of feasibility (e.g., consent rate, percentage of consenting participants who see the physiotherapist first who also request to see the physician or nurse practitioner). Due to the nature of the intervention, patient participants and HCPs will not be blinded in the fully powered trial. Since the primary outcome measures are self-report measures (i.e., the participant is the assessor), the outcome assessor is similarly not planned to be blinded.

### APP role training

The core competencies informing the training of the APP were identified through a series of focus groups with an interprofessional panel of team members and patient participants using a nominal group technique and follow-up Delphi process (results not yet published) [53]. The PT recruited for the role in this feasibility study has several years of experience working with the Chronic Pain Clinic. To obtain the competencies for this role, the PT completed a training program outside of his normal responsibilities. The training included spending time shadowing, providing shared care, and practicing competencies with members of each profession from the interprofessional Chronic Pain Clinic team. The medical director of the Chronic Pain Clinic, one of the team members that frequently performs the initial assessment and development of the care plan, evaluated the PT on the competencies established through our focus groups (see competency checklist — Additional file 2). As a competency-based process, the training focused on the development of competencies, emphasized the abilities of the APP (not just knowledge), de-emphasized time-based training (allowing flexibility to achieve the competencies at different rates), and was learner centered [54]. As a competency-based training program, the training for the APP role was not meant to be standardized; rather, readiness to fulfill the APP role was assessed by the medical director.

### Usual care

Usual care at the KHSC Chronic Pain Clinic incorporates a physician- or nurse practitioner-led model, whereby patient participants are referred to the clinic to participate in an initial assessment by a physician or nurse practitioner who will make recommendations for the plan of care (e.g., urgent or emergent referrals for further investigation, medication management, referrals to allied HCPs, interventional procedures, or group-based treatments). Under this model of care, the physician or nurse practitioner may recommend the patient sees other members within the interprofessional Chronic Pain Clinic or as an outpatient in the community. While the physician or nurse practitioner collaborates with and draws on the expertise of the various members of the Chronic Pain Clinic team, they typically maintain the responsibility for directing and coordinating care until the patient is discharged from the Chronic Pain Clinic or a referral is made to another HCP to coordinate care.

### Intervention

The trained APP for chronic pain will provide an initial intake assessment, instead of the physician or nurse practitioner, and make care recommendations for patient participants, including what interprofessional team members will be involved in the participant’s care. In alignment with practice guidelines [55–57], the APP will approach the assessment with a biopsychosocial approach [29]. The assessment will include taking a thorough history; screening for pathologies contributing to pain; physical examination; using evidence-based screening tools to identify comorbid health conditions that require specific care (e.g., depression, addiction, post-traumatic stress); using validated tools to identify psychosocial risk factors associated with ongoing pain and disability; measuring pain severity and interference; and providing education on pain, prognosis, clinical processes, and team members. The APP will provide recommendations to the participant and healthcare team based on the assessment findings (e.g., urgent or emergent referrals for further investigation, medication management, referrals to allied HCPs, interventional procedures, or group-based treatments). Like the usual care model, the APP will collaborate with the wider team; however, the APP will maintain the overall responsibility for directing and coordinating the care until the participant is discharged from the Chronic Pain Clinic or a referral is made to another HCP to coordinate care. The APP will not be practicing in an extended scope role and, therefore, is fully autonomous and will not require delegation or transfer of responsibilities from a physician on the team.

### Evaluation and outcome measures

Participants will be asked to complete an online survey through a secure online data collection platform [Qualtrics, Provo, UT, USA [58]] to obtain baseline measures prior to their visit with the APP. Follow-up assessments will be collected electronically (or in person or by phone if requested by the participant) at 3, 6, 9, and 12 months after the initial assessment. If the participant does not complete the electronic survey, follow-up emails and phone calls will be made.

### Baseline measures

To describe the study population, the following data will be collected at baseline through the survey: age, gender, biological sex, education, identification as a member of a racialized group, identification as indigenous (First Nations, Inuit, Métis), duration and location of pain, comorbid conditions, current medications, work status, rurality, and annual household income. Comorbidities will be measured using the Self-Administered Comorbidity Questionnaire [59]; respondents select relevant comorbidities from a list of specific problems (with three optional, open-ended conditions), whether they receive treatment for the condition, and whether the problem limits their activities.

### Feasibility of trial design

#### Recruitment rate (patient participants/week)

The full trial will be deemed feasible, as planned, if we are able to recruit 40 participants over eight weeks (5/week). Additional sites or duration will be planned if this recruitment rate is not achieved.

#### Assessment completion

Given the longitudinal nature of this study, particular attention needs to be paid to the completeness of the outcome measures, in this case, completion of the surveys at each time point. We have determined that acceptable completeness will be satisfied if > 80% of all assessment items are completed across all follow-up time points.

#### Duration of survey completion

We will consider mean times for the completion of baseline and follow-up surveys of less than 60 min each to be acceptable. Completion times will be measured using the start and end times of the surveys.

#### Attrition rate

Retention will be evaluated by rate of attrition. An attrition rate of < 20% at 12-month follow-up is considered indicative of feasibility. Research has suggested that > 20% attrition threatens study validity [60].

### Feasibility of implementing the APP model of care

#### Care provided

Health care provided at the Chronic Pain Clinic will be collected from the electronic medical record (EMR), including prescriptions for medications, interventions delivered, requisitions for diagnostic imaging, referrals to other HCPs, visits to HCPs from each professional background, and relevant notes to employers or insurers. For the feasibility study, the collection of this information will be used to reduce uncertainty around the care provided as part of the APP model of care in the full trial and the capacity of the APP to develop a plan of care and appropriately integrate other team members where needed. The model will be considered feasible if the APP can successfully fulfill the role for at least 80% of participants without having to transfer care to the physician or nurse practitioner as the responsible provider. If more than 20% of participants request transfer of care to the physician or nurse practitioner, or the APP decides a transfer of care to the physician or nurse practitioner is needed, modifications to the initial RPN triage process will be made in preparation for the full trial.

#### Treatment fidelity

Treatment fidelity will be determined using an APP-reported fidelity checklist (Additional file 3). This will allow us to evaluate consistency of the intervention with the proposed initial assessment and care plan methods. An acceptable level of fidelity will be considered with completion of 100% of screening for pathological concerns (for participant safety) and > 80% for all other assessment items.

### Assessment of contextual factors that may influence implementation

Contextual factors that may influence implementation will be evaluated using semi-structured interviews with patient participants and HCPs using an interpretive description approach [61]. Contextual factors that will be explored will include patient participant (e.g., acceptability, satisfaction with the model of care), provider (e.g., trust, perceived value), and clinic level (e.g., perceived influence on clinic processes) barriers and facilitators. We anticipate that the findings from these qualitative interviews will lead to strategies for refining the model of care and associated processes prior to a fully powered trial. We will recruit 10–12 patient participants using purposive sampling with the aim of ensuring maximum variation in age, sex, gender, and duration and location of pain. We will recruit 5–10 HCPs using a convenience sample of health professionals within the Chronic Pain Clinic team. We will aim to include at least one member of each health professional group represented within the team (i.e., medicine, nursing, nurse practitioner, occupational therapy, psychology, physiotherapy, and social work). The interviews will be conducted by a member of the research team over Zoom or through phone call and will last approximately 30 to 60 min. An interview guide will be developed by the interdisciplinary research team and informed by previous studies aimed to explore contextual factors related to the implementation of new advanced practice roles [42, 62]. The qualitative interviews will be recorded and transcribed verbatim. To ensure confidentiality, the data from the interviews will be de-identified before analysis. Data analysis will be conducted using NVivo data management software through a reflexive thematic analysis approach [63]. To ensure rigor, two independent researchers will code the data and conduct the analysis iteratively to ensure reliability of the coding and reduce researcher bias. Researchers will practice reflexivity by maintaining a reflexivity journal and participating in frequent reflexive dialogue among research team members. The researchers will keep an audit trail of the coding process and how themes were generated to ensure transparency.

Patient participant interviews will be completed approximately 1–2 months after their initial visit with the APP, and HCP interviews will occur 1–2 months after the last patient participant has been assessed by the APP. This recall period was considered appropriate given the complexity and context of the questions to be asked for this element of the study [64]. Waiting for all participants to be assessed allows HCPs the full opportunity to be exposed to the APP model of care. The interviewer will ask participants to identify barriers and facilitators to the implementation of the APP model of care and strategies for refining the model of care for the full trial. HCP participants will be asked about the impact on clinical processes and patient care.

Descriptive details regarding the interviewed patient participants will include age, sex, gender, living status, annual household income, education, location of pain, duration of pain, and primary diagnosis causing chronic pain. For the HCPs interviewed, descriptions of their health profession, number of years practicing, and number of years working at the KHSC Chronic Pain Clinic will be described.

### Assessment of outcomes in preparation for the full trial

As part of assessing the feasibility of collecting all assessment data, we will collect all patient participant health outcomes, care provided, flow through the Chronic Pain Clinic, and data for the planned cost utility analysis to inform our intended methods for a fully powered clinical trial. Unless indicated otherwise, these outcomes will be collected using Qualtrics and will be collected at all time points (baseline, 3, 6, 9, and 12 months).

### Participant health outcomes

*Pain severity* and *pain interference* will be captured using the Brief Pain Inventory (BPI) pain severity subscale and pain interference subscale. Both subscales use a numeric rating scale from 0 to 10, with higher scores indicating greater pain or interference, respectively. The BPI shows good reliability and validity for its use among individuals with chronic pain [65] and is the planned primary patient participant health outcome for a full trial.

*Health-related quality of life* will be examined using the EuroQOL-5D-5L [66]. This measure will be used in a cost-utility analysis with the aid of corresponding calculated quality-adjusted life years (QALYs) [67–69]. A value set derived for the Canadian context will be applied to this outcome measure [70].

*Psychosocial risk factors for pain* that are prognostic indicators for outcomes of chronic pain management will be evaluated using the Pain Catastrophizing Scale [71, 72], Tampa Scale of Kinesiophobia [73], and Pain Self-Efficacy Questionnaire [74].

*Self-reported rating of change* will be measured, at follow-up time points, using a global rating of change scale (GROC). The GROC will use an 11-point scale (− 5 to + 5), with negative values representing a perceived worsening and positive values a perceived improvement in functional abilities. The GROC is recommended as a valid self-reported measure of change [75, 76].

*Satisfaction with care* will be assessed using an 11-point scale (0 to 10) at all follow-up time points [77, 78]. Low scores demonstrate dissatisfaction, and positive score suggests satisfaction with care.

*Adverse events* will be monitored and reported using an adverse events questionnaire that is in accordance with event reporting guidelines [51, 79]. This section of the survey requests information regarding the type of adverse event experienced, how long the event lasted, how bothersome the event was (0–10 scale), and what the participant thought caused the event.

### Flow of participants through the Chronic Pain Clinic

One of the goals of the APP model of care is to improve patient flow through interprofessional chronic pain clinics. We hypothesize that the APP model of care may improve patient flow by triaging appropriate participants to care from interprofessional team members other than the physicians (anesthesiologists or other pain specialists) and nurse practitioners directly to those services. There are many services within chronic pain clinics that have much shorter wait-lists (e.g., interprofessional self-management programs, physiotherapy, occupational therapy, social work). Triaging participants appropriate to these services earlier would improve the flow of patients to those services and potentially improve the flow for those who require the specialized services of the anaesthesiologists or nurse practitioners (e.g., medication management or procedures such as injections) by off-loading some of the initial assessment responsibilities from these team members; therefore, we will assess the proportion of participants who are triaged to health services without individual appointments with physicians or nurse practitioners as a feasibility measure for the potential for this model of care to impact patient flow in a future trial.

### Cost-utility analysis

A cost-utility analysis of the APP model of care compared to the physician-led model is planned for the full trial using a societal perspective [80, 81]. Healthcare utilization measures will be captured in the survey and used to determine costs. These utilization measures will include emergency department visits, overnight hospitalizations, diagnostic imaging, surgical interventions, pain injections/procedures, primary care visits (including walk-in clinic visits), specialist physician visits, medications used, physiotherapy, occupational therapy, chiropractic appointments, self-care assistance, and other health provider visits (e.g., massage therapy, social worker visits, psychologist appointments). The survey will capture these measures using specific, pointed questions; for example, “Over the past 12 weeks, how many times did you visit the emergency room for your chronic pain?”.

Direct healthcare costs will be calculated using the Ontario Ministry of Health and Long-Term Care Schedule of Benefits for publicly funded health services and the Ontario Drug Benefit formulary for medication costs. For private healthcare services (e.g., PT in the community), the mean cost for the services in Kingston, Ontario, will be used.

Indirect or non-healthcare costs will be restricted to loss of productivity using a human capital approach. A dollar value will be assigned to time lost from paid employment (part-time, full-time, and self-employment) based on the mean wage in Ontario, according to Statistics Canada. The minimum wage in Ontario will be used to assign a value to time lost from volunteering, caregiving, or homemaking activities. Participants will also be able to report, in the survey, any other out-of-pocket expenses they may have incurred as a result of their chronic pain.

### Alignment of care provided with quality standards for chronic pain management

Ontario Health has provided key statements describing quality standards for the management of chronic pain [9]. We have operationalized these standards (Additional file 4) as a means of evaluating the APP model of care and, in the full trial, comparing it to the usual physician- or nurse practitioner-led care. Checklists related to quality and process indictors will be used to measure and describe adherence to all quality standards based on available details in the participant’s medical chart across all visits.

### Data collection and management

All survey responses will be captured in Qualtrics, and respondents will be assigned a unique study identification number. Participants will be instructed on how to complete the surveys by a trained research assistant. At study completion, all survey responses will be exported directly from Qualtrics to encrypted and password-protected spreadsheets and stored securely in Queen’s Microsoft OneDrive for Business. Contact information (telephone number and email) are required to contact the participants for study-related data collection and survey distribution. Full name and age will be required to access the participant’s EMR. All information collected from the EMR, along with the master log linking the study identification numbers to the participants, will be entered into encrypted and password-protected files and stored securely in Queen’s Microsoft OneDrive for Business. Personal information will be accessible only to study investigators and research staff and will be deleted at the completion of the data collection period. Audio recordings for the qualitative interviews will be transcribed and stored securely in OneDrive. The audio recordings will be deleted immediately following transcription. All de-identified data will be deleted after a 10-year storage period.

### Analysis

Descriptive statistics for all relevant baseline measures and outcomes will be reported, and decisions will be made regarding study feasibility based on the thresholds described under each feasibility outcome (e.g., < 20% attrition, > 80% of assessment items completed, and > 80% of items performed on the treatment fidelity checklist). Means (standard deviation) or medians (interquartile range) for continuous variables and frequencies (percent) for categorical variables will be presented. Quality indicator calculations are presented in the quality standards operationalization table (Additional file 4) and will be presented accordingly for each quality statement.

Health utilization measures will be presented as counts, and costs will first be calculated as the product of resource utilization and the corresponding unit cost. Summing these costs will provide the total costs and will be further assessed at each follow-up interval. The mean cost for the entire study period, as well as the mean cost for each time interval, will be presented.

We will document challenges in the collection and analysis of all data to inform the plan for the full trial where we intend to perform a complete assessment of adherence to quality standards and cost utility analysis.

The transcribed interviews will be analyzed using an interpretive description approach [61] to generate clinically relevant themes. Thematic analysis [63] will be performed by two investigators who will independently code the transcripts. As described by Braun and Clarke [63], the thematic analysis process will include familiarization with the data, production of initial codes, finding and reviewing potential themes, naming themes, and producing a report of the analysis. Identified themes will be used to describe patterns in patient participant and HCP responses to translate their perspectives into meaningful representations of the experience with the APP model of care as they relate to the acceptability of the APP role; barriers, facilitators, and strategies for refining model implementation; and perceived impact on clinical processes and outcomes.

### Protocol amendments

Any changes to the feasibility protocol will be communicated by appending the trial registry at ClinicalTrials.gov and reported in the feasibility trial report. Investigators and participants will be communicated with as appropriate, depending on the changes.

## Discussion

The complex nature of a longitudinal interprofessional clinical trial requires the coordination of interprofessional HCPs, integration of supportive and administrative staff, and the recruitment and retention of participants over a relatively long period of time. These factors make it essential to take responsible and calculated steps to ensure the success of such a large-scale endeavor that utilizes an extensive number of resources. This study is intended to demonstrate the feasibility of integrating an APP role within established chronic pain clinical settings. The study will also help inform and refine our methods as needed for a future full-scale trial. For example, the process of collecting chart information to describe adherence to quality standards may present opportunities to improve the operational definition of each component. Also, questions on the survey can be modified if ambiguity arises as a result of unexpected participant responses. The qualitative interviews offer us the opportunity to evaluate practical experience, from patient participants and HCPs, which can allow us to improve upon our methods for a full trial. The results of this feasibility study will be published in a peer-reviewed journal, and targeted summaries of results will be communicated to participants and interprofessional team members at the Chronic Pain Clinic.

The objectives of the full study will be to describe patient participant health outcomes, care provided to participants, chronic pain clinic flow, the delivery of quality standard care, and cost utility of the APP model in comparison to the usual physician- or nurse practitioner-led care in Canada. The results are intended to inform stakeholders who are considering the transition of including an APP for the initial assessment and development of care plan role within their chronic pain clinical setting. The study builds on a growing body of evidence on the role of APPs in other settings, including orthopedic clinics and emergency departments. Successful outcomes may encourage the ubiquitous use of this model of care in an effort to provide effective and efficient interprofessional patient care for individuals experiencing chronic pain who often have complex health needs.

## Supplementary Information

**Additional file 1.** Modified SPIRIT checklist.

**Additional file 2.** Advanced Practice Physiotherapist Competency Evaluation Form.

**Additional file 3.** Advanced practice physiotherapist fidelity checklist.

**Additional file 4.** Quality of care for patients with chronic pain.

## Data Availability

Not applicable.

## Abbreviations

APP: Advanced practice physiotherapist
BPI: Brief Pain Index
EMR: Electronic medical record
GROC: Global rating of change
HCP: Healthcare provider
KHSC: Kingston Health Sciences Centre
MRC: Medical Research Council
PT: Physiotherapist
QALY: Quality-adjusted life years
RPN: Registered practical nurse
