# Supplementary material for: Assessing the feasibility of a clinical trial to evaluate an advanced practice physiotherapy model of care in chronic pain management: a feasibility study
Source: Pilot Feasibility Stud. 2023 Jul 17;9:125. doi: 10.1186/s40814-023-01352-9 (PMC10351139; doi:10.1186/s40814-023-01352-9)
Supplement: Supplementary file 2 — Additional file 2. Advanced Practice Physiotherapist Competency Evaluation Form. [file 40814_2023_1352_MOESM2_ESM.docx]

**Additional file 2 - Advanced Practice Physiotherapist Competency Evaluation Form**

APP candidate: ________________________________________ Date: ___________________

Assessor (name and title): ________________________________

Medical director: _______________________________________

**Type of Evidence:** Self-assessment (SA), Written responses (WR), Oral appraisal (OA), Documentary evidence (DE), Workplace observation (WO), Case-based presentation (CBP), Qualification/training record (Q/T), Recognition of prior learning evidence (RPL), Portfolio (PF), Performance appraisal (PA), Other (please specify)

| **Elements of the APP competency** | **Performance rating scale** | | | | | | **Type of Evidence** |
| --- | --- | --- | --- | --- | --- | --- | --- |
|  | **X or N/A** | **Dependent** | **Marginal** | **Assisted** | **Supervision** | **Independent** |  |
| 1. **Use an evidence-based approach to practice**   Integrate evidence, patient perspective, and clinical experience into clinical decision making. |  |  |  |  |  |  |  |
| 1. **Communicate effectively with the patient**   Listen actively to patients and use effective verbal and non-verbal communication while completing an assessment, educating patients, communicating a diagnosis, and recommending and evaluating the care plan. The APP adapts communication to meet the needs of patients, their families, and their substitute decision makers. |  |  |  |  |  |  |  |
| 1. **Perform a comprehensive assessment**   Complete a comprehensive assessment of the patient’s pain experience, contributing factors, and impacts to determine which health professionals to involve in the patient’s care. This includes a history, validated questionnaires, and a physical examination. The assessment screens for biological, psychological, and social factors; past or current treatment history; and comorbid health conditions that impact pain, quality of life, or treatment response. The assessment should help the APP understand the patient’s goals and expectations for pain management and assess patient’s safety. |  |  |  |  |  |  |  |
| 1. **Determine a pain-related diagnosis(es)**   Integrates all assessment findings to formulate a pain related diagnosis or classification that identifies potential impairments, limitations in activities and restrictions in participation and factors influencing pain and functioning positively or negatively. |  |  |  |  |  |  |  |
| 1. **Develop therapeutic relationships**   Develop therapeutic relationships based on mutual trust and compassion that support patient’s autonomy. |  |  |  |  |  |  |  |
| 1. **Provide appropriate care**   Recommends and collaborates on the implementation of a care plan that may include referral to appropriate healthcare providers or community resources within and external to chronic pain clinic team. The APP provides physiotherapy treatment, self-management support, education, and reassurance when appropriate. |  |  |  |  |  |  |  |
| 1. **Support patients through transition in care**   Coordinate and support the continuity of care as the patient transitions between settings and between health professionals within or outside of the chronic pain clinic |  |  |  |  |  |  |  |
| 1. **Collaborate with members of interprofessional team**   Collaborate with patients and families, members of the chronic pain clinic team, healthcare providers external to the team, and community service providers to provide integrated health services. |  |  |  |  |  |  |  |
| 1. **Advocate for the needs of the patient**   Advocate for accessibility of health and social services that meet the patient’s needs |  |  |  |  |  |  |  |
| 1. **Use a reflective approach to practice**   Integrate self-reflection, peer and patient feedback to continuously improve quality and appropriateness of care. |  |  |  |  |  |  |  |

**Description of the performance rating scale**

| **Scale labels** | **Standard of procedure** | | **Quality of performance** | **Level of assistance required** |
| --- | --- | --- | --- | --- |
| Independent (I) | Safe Accurate | Achieved intended outcome Behaviour is appropriate to context | Proficient Confident Expedient | Independent (I) |
| Supervised (S) | Safe Accurate | Achieved intended outcome Behaviour is appropriate to context | Proficient Confident Reasonably expedient | Supervised (S) |
| Assisted (A) | Safe Accurate | Achieved most objectives for intended outcome Behaviour generally appropriate to context | Proficient throughout most of the performance when assisted | Assisted (A) |
| Marginal (M) | Safe only with guidance Not completely accurate | Incomplete achievement of intended outcome | Unskilled Inefficient | Marginal (M) |
| Dependent (D) | Unsafe | Unable to demonstrate behaviour Lack of insight into behaviour appropriate to context | Unskilled Unable to demonstrate behaviour/procedure | Continuous verbal and physical directive cues |
| X | Not observed |  |  |  |
