# Supplementary material for: Assessing the feasibility of a clinical trial to evaluate an advanced practice physiotherapy model of care in chronic pain management: a feasibility study
Source: Pilot Feasibility Stud. 2023 Jul 17;9:125. doi: 10.1186/s40814-023-01352-9 (PMC10351139; doi:10.1186/s40814-023-01352-9)
Supplement: Supplementary file 3 — Additional file 3. Advanced practice physiotherapist fidelity checklist. [file 40814_2023_1352_MOESM3_ESM.docx]

**Additional file 3 - Advanced practice physiotherapist fidelity checklist – initial assessment**

| **Participant ID** | **History** | **Screening for pathology^*^** | **Screening for psychosocial factors^†^** | **Physical examination** | **Screening tools to identify comorbid conditions that require specific care** | **Sets collaborative goals with participant** | **Education provided on clinic processes and team members** | **Education regarding pain prognosis** | **Proposed management plan discussed** |
| --- | --- | --- | --- | --- | --- | --- | --- | --- | --- |
|  |  |  |  |  |  |  |  |  |  |
|  |  |  |  |  |  |  |  |  |  |
|  |  |  |  |  |  |  |  |  |  |

Place an “X” in the cells to indicate that the item was completed

^*^Pathology (or “red flag”) screening involves screening for pathology contributing to the individual’s pain. Includes malignancy, cardiovascular, gastrointestinal, neurological, or other concerning signs and symptoms (1).

^†^Psychosocial factors (or “yellow flags”) screening involves screening for psychosocial barriers to recovery, including fear of movement behaviours, social withdrawal, low mood, excessive rest, work compensation conflicts, lack of social support (2, 3).

1. Magee DJ. Orthopedic Physical Assessment. Enhanced Edition. 4th ed. Philadelphia: Saunders; 2006.

2. Moffett J, McLean S. The role of physiotherapy in the management of non-specific back pain and neck pain. Rheumatology. 2005;45(4):371-8.

3. Kendall NAS, Linton SJ, Main CJ. Guide to Assessing Psycho-social Yellow Flags in Acute Low Back Pain: Risk Factors for Long-Term Disability and Work Loss. Wellington, New Zealand: Accident Compensation Corporation and the New Zealand Guidelines Group; 2004.
