# Supplementary material for: Assessing the feasibility of a clinical trial to evaluate an advanced practice physiotherapy model of care in chronic pain management: a feasibility study
Source: Pilot Feasibility Stud. 2023 Jul 17;9:125. doi: 10.1186/s40814-023-01352-9 (PMC10351139; doi:10.1186/s40814-023-01352-9)
Supplement: Supplementary file 4 — Additional file 4. Quality of care for patients with chronic pain. [file 40814_2023_1352_MOESM4_ESM.docx]

**Additional file 4 – Alignment of care provided with Ontario Health Quality standards for chronic pain care** (1)

| **Quality standards** | **Description in Quality Standard** | **Indicators proposed in the Quality Standard** | **Operational definition of this indicator within our study** |
| --- | --- | --- | --- |
| Quality Statement 1: Comprehensive Assessment | **Comprehensive Assessment**: Description of the pain condition; signs of serious underlying pathology; medication use and effectiveness for pain and function; functional status, or attendance at school for children and youth; sleep patterns; past and current mental health status; food security, nutritional status, or deficiencies; psychosocial history and issues; past and current substance use, a family history of substance use; family history of chronic pain; past pain management and coping strategies and their effects; beliefs, knowledge, and level of understanding of pain and pain management; assessment of cognitive status, frailty, and impairment (physical or cognitive); physical examination.  Functional status: refers to a person’s ability to perform activities of daily living, including work, play, and socialization (should be performed using a validated assessment tool. | **Process Indicators:**  **Percentage of people with chronic pain who receive a comprehensive assessment**  • Denominator: total number of people with chronic pain  • Numerator: number of people in the denominator who receive a comprehensive assessment  **Number of days between when people report chronic pain symptoms and when they have a completed comprehensive assessment**  • Calculation: can be measured as mean, median, or distribution of the wait time (in days) between when people report chronic pain symptoms and when they have a completed comprehensive assessment | **Quality indicator 1:** % of patients with complete comprehensive assessments = (# complete assessments / total # assessments) x 100  Definition of a complete comprehensive assessment = completion of ALL components listed below are documented in the electronic medical record   - - Percentage completion of each component will further be presented individually   Completion of each component listed below will be indicated as yes/no in intake forms or chart   \| Component \| Intake forms \| Found in chart notes \| \| --- \| --- \| --- \| \| Pain description \| Validated tools used to assess pain description (e.g., BPI pain severity subscale, McGill Pain Questionnaire, NRS) \| Unidimensional measures (e.g. NRS, VRS, VAS)  Written descriptors \| \| Screening for serious pathology \| Intake screening questions \| Evidence of red flag screening \| \| Functional status \| Validated tools for function (e.g., RMDQ, BPI pain interference subscale, FIM – motor subscale, PROMIS pain interference scale) \| - Sit-to-stand  - Gait analysis  - Balance (including Berg)  - Functional reach  - 6-minute walk  - Timed up and go  - ROM, strength  - Specific task assessment  - Posture assessment \| \| Medication review \| Listed on referral package or intake forms \| Evidence of review in notes \| \| Sleep patterns \| Validated tools \| Assessment of sleep patterns recorded in health professional notes \| \| Mental health status \| Validated tools for mental health screening or assessment  Completed mental health assessment questions on intake forms \| Assessment of mental health recorded in health professional notes \| \| Food security or nutritional status \| Completed food security questions on intake forms \| Assessment of food security recorded in health professional notes \| \| Psychosocial history \| Validated tool (e.g., PCS, BDI, PHQ-9, CSQ) \| Record of discussing the following:  - Emotions  - Relationships  - Attitudes/beliefs \| \| Substance use \| Completed substance use questions on intake forms \| Record of discussing substance use \| \| Family history of chronic pain \| Completed family history questions on intake forms \| Record of discussing family history of chronic pain \| \| Past pain management and coping strategies and their effects \| Completed pain management and coping strategies questions on intake forms \| Record of discussing past pain management and coping strategies \| \| Beliefs, knowledge, level of understanding of pain \| Completed questions or assessment tools to identify knowledge or beliefs about pain \| Record of discussing beliefs, knowledge, and level of understanding of pain \| \| Assessment of cognitive status, frailty, impairment (physical or cognitive) \| Validated tools (e.g., FIM – cognition subscale, MOCA, MMSE) \| Evidence in notes of assessment of cognitive status (e.g. orientation, recall), frailty (e.g. Fried’s phenotype method, SPPB) \| \| Physical examination \| N/A \| Evidence of physical examination in chart \| |
| Quality Statement 2: Setting Goals for Pain Management and Function | **Goals:** **SMART** (set specific, measurable, achievable, relevant, and time-limited) goals that may include pain management, activities of daily life, sleep, mental health  **Evaluate regularly**  Management goals for pain and function should be documented and monitored over time. Progress toward goals should be reassessed at least every 3 months, or more frequently, based on nature of the specific goals developed | **Process Indicator:**  **Percentage of people with chronic pain who have documented goals for pain management and functional improvement**  • Denominator: total number of people with chronic pain  • Numerator: number of people in the denominator who have documented goals for:  - Pain management  - Functional improvement | **Quality indicator 2a:** % of patients with documented goals in their electronic medical record = (# with documented goals / total # assessments) x 100  **Quality indicator 2b:** % of patients with documented goal revaluation within three months of initial assessment = (# with documented goals revaluation / total # assessments) x 100 |
| Quality Statement 3: Supported Self-Management and Education | **Education:** Can be provided in person, written, or online provided by a health care professional, trained peer educator, or other trained person  **Education should include evidence-based information on**: The pain condition; pain mechanisms, pathologies, and the processes of healthy normal functioning; the physiology of pain, including differences between acute and chronic pain; the psychology of pain, including fear and avoidance, stress, and depression; the benefits of physical activity and exercise; recommended physical, psychological, pharmacological, and self-management interventions and strategies; healthy eating, nutrition, and weight management; the risks of substance use, including cigarettes, alcohol, and opioids; the management of other concurrent chronic conditions; evidence of potential benefits and harms for all therapies for chronic pain, where they exist.  **Self-management:** Self management programs can be group or individual based. | **Process Indicators:**  **Percentage of people with chronic pain who receive education on chronic pain**  • Denominator: total number of people with chronic pain  • Numerator: number of people in the denominator who receive education on chronic pain  **Percentage of people with chronic pain who access self-management resources or programs**  • Denominator: total number of people with chronic pain  • Numerator: number of people in the denominator who access self-management resources or programs  **Outcome Indicator:**  **Percentage of people who report feeling better able to cope with their pain after receiving education or after accessing self-management programs**  • Denominator: total number of people with chronic pain who receive education or who access a self-management program  • Numerator: number of people in the denominator who feel better able to cope with their pain | **Quality indicator 3a:** % of patients who received education on chronic pain = (# with documented education on chronic pain / total # assessments) x 100  **Quality indicator 3b:** % of patients who accessed to self-management program = (#% who accessed to self-management program / total # assessments) x  **Quality indicator 3c**: % of patients with improvement from self-management or education = (# with documentation of improvement from self-management or education / total # who access received education or accessed self-management program) x 100 |
| Quality Statement 4: Physical Activity | **Physical activity:** Any activity that involves bodily movement, done as part of leisure, recreation, work, play, active transportation, or household tasks. It is different from therapeutic exercise, which is a type of physical activity that is planned, structured, repetitive, and designed to improve or maintain one or more components of physical fitness (3). | **Process Indicators:**  **Percentage of people with chronic pain who receive information from their health care professional about engaging in regular physical activity**  • Denominator: total number of people with chronic pain  • Numerator: number of people in the denominator who receive information from their health care professional about engaging in regular physical activity  **Percentage of people with chronic pain who report participating in daily physical activity**  • Denominator: total number of people with chronic pain  • Numerator: number of people in the denominator who report participating in daily physical activity | **Quality indicator 4:** % of patients who receive education on or have a documented discussion about physical activities they are already engaged in = (# who receive education on or are engaged in physical activity / total # with chronic pain) x 100  Evidence for education on or documented discussion on activities the patient is already engaging in:   - Engagement in leisure activity - Recreational activity - Walking - Cycling (e.g., cycling to work, means of transportation) - Gardening - Other   Note: engagement includes activities listed that the patient is already participating in. |
| Quality Statement 5: Physically Based Interventions | **Types of physically based interventions to be considered in the management of chronic pain include**:  • Manual therapy for short-term relief of pain in chronic low back pain  • Transcutaneous electrical nerve stimulation (TENS) for chronic pain. Either low- or high-frequency TENS can be used  • Low-level laser therapy for chronic low back pain  • Breathing activities  Other interventions to consider include postural correction, walking aides, and environmental changes to the home or worksite | **Process Indicator:**  **Percentage of people with chronic pain who receive physically based interventions for chronic pain**  • Denominator: total number of people with chronic pain  • Numerator: number of people in the denominator who receive physically based interventions for chronic pain | **Quality indicator 5:** % of patients who receive physically based interventions = (# who receive physically based interventions / total # with chronic pain) x 100  Evidence for receiving any of the following:   - Manual therapy (for chronic low back pain only – not checked if performed for other pain conditions) - Transcutaneous electrical nerve stimulation (TENS) - Low-level laser therapy (for chronic low back pain only - not checked if performed for other pain conditions) - Breathing exercises - Postural correction - Walking aides - Ergonomic/environmental changes to work or home |
| Quality Statement 6: Therapeutic Exercise | **Therapeutic exercise:** Exercise as a therapeutic intervention is defined as a structured, repetitive, physical activity designed to improve or maintain physical fitness and maintain or increase range of motion (3). It is recommended or prescribed by a health care professional and can be facilitated or implemented by another trained person | **Process Indicator:**  **Percentage of people with chronic pain who participate in therapeutic exercise**  • Denominator: total number of people with chronic pain  • Numerator: number of people in the denominator who participate in therapeutic exercise | **Quality indicator 6:** % of patients who receive education or are engaged in therapeutic exercise = (# who receive education or are engaged in therapeutic exercise / total # with chronic pain) x 100  Evidence of prescription for or engagement in therapeutic exercise includes any of the following:   - Range of motion exercises - Strengthening exercises - Stretching exercises - Instructed exercise classes (e.g., aquatic therapy, yoga, aerobic class) - Graded, structured, and planned physical activity (e.g., walking, running, cycling) with the goal of improving physical fitness   Note: the presence of dosage elements [frequency, intensity, time, type, volume, and/or progression (4) components] meets the definition of exercise. |
| Quality Statement 7: Pharmacotherapy | **Non-opioid pharmacotherapy**: Non-opioid medications for chronic pain include several types of medications. Non-opioid medications to consider for the management of chronic pain include the following   - NSAIDs(For chronic non-specific low back pain). - Anticonvulsants (Gabapentin, Pregabalin, Carbamazepine), - Antidepressants (Tricyclic antidepressants such as amitriptyline, nortriptyline, or imipramine), Amitriptyline, Duloxetine, Fluoxetine - Acetaminophen - Topicals - Cannabinoids (includes cannabis) | **Process Indicators:**  **Percentage of people with chronic pain who receive non opioid pharmacotherapy as first-line pharmacological treatment for chronic pain**  • Denominator: total number of people with chronic pain who are prescribed pharmacotherapy to control their pain  • Numerator: number of people in the denominator who receive non-opioid pharmacotherapy as first-line pharmacological treatment  **Percentage of people with chronic pain who receive pharmacological treatment to control their pain whose medications are reviewed for effectiveness in pain management and meeting goals**  • Denominator: total number of people with chronic pain who receive pharmacological treatment to control their pain  • Numerator: number of people in the denominator whose medications are reviewed for effectiveness in pain management and meeting goals | **Quality indicator 7a:** % of patients who receive non-opioid pharmacotherapy as first-line pharmacological treatment = (# who non-opioid as first-line treatment^a^ / # who are prescribed pharmacotherapy treatment^b^) x 100  ^a^No evidence in chart of current opioid medication (among those in ^b^)  ^b^Being prescribed pharmacotherapy of any kind  **Quality indicator 7b:** % of patients who receive pharmacological treatment whose medications are reviewed = (# whose medications are reviewed / total # who receive pharmacotherapy treatment) x 100 |
| Quality Statement 8: Psychologically Based Interventions | **Psychological assessment:** Assessment should include a discussion about ability to cope with chronic pain symptoms and any impacts on mood and mental health. Assessment should also be offered for the diagnosis of depression or anxiety.  **Psychologically based interventions:**   - Acceptance and commitment therapy - Cognitive behavioural therapy - Dialectical behavioural therapy - Mindfulness-based interventions   Progressive relaxation or electromyographic biofeedback | **Process Indicators:**  **Percentage of people with chronic pain who receive psychological assessment**  • Denominator: total number of people with chronic pain  • Numerator: number of people in the denominator who receive psychological assessment  **Percentage of people with chronic pain who receive appropriate psychologically based interventions**  • Denominator: total number of people with chronic pain  • Numerator: number of people in the denominator who receive appropriate psychologically based interventions | **Quality indicator 8a:** % of patients who receive psychological assessment = (# who receive psychological assessment / total # with chronic pain) x 100  **Quality indicator 8b:** % of patients who receive psychological oriented intervention = (# who receive psychological intervention / total # with chronic pain) x 100 |
| Quality Statement 9: Psychosocial Supports | **Accessibility needs** include physical, developmental, intellectual, learning, visual, auditory, and emotional supports and services to make a health care environment or treatment accessible. Where appropriate, it may also include access to language interpretation services.  **Cultural needs** relate to the beliefs and preferences that come from one’s social and ethnic identities. Cultural needs may be related to linguistic needs, health beliefs and behaviours, traditions, rituals, or cultural barriers to accessing health care.    **Psychosocial support** involves developmentally and age-appropriate care related to a person’s state of mental, emotional, social, cultural, and spiritual well-being. It can also include cognitive therapies or strategies such as those related to memory, attention, executive functioning, or fatigue**.**  **Social needs** relate to a person’s relationships with their family, community, and network (friends, acquaintances, and coworkers). Social needs may relate to family structure and location; family dynamics; communication; social and cultural networks; perceived social support; work and school settings; finances; sexuality; intimacy; living arrangements; caregiver availability; medical decision making; access to transportation, medications, equipment, and nutrition; community resources; or legal issues.  **Spiritual needs** relate to “the way individuals seek and express meaning and purpose, and the way they experience their connectedness to the moment, to self, to others, to nature, and to the significant or sacred.” Spiritual needs may include religious practices or philosophical reflection | **Percentage of people with chronic pain who receive psychosocial supports**  • Denominator: total number of people with chronic pain  • Numerator: number of people in the denominator who receive psychosocial supports | **Quality indicator 9:** % of patients who receive psychosocial supports = (# who receive psychosocial support / total # with chronic pain) x 100  Evidence of psychosocial support in the chart addressing any of the following:   - Accessibility to health services - Cultural needs (language, health beliefs and behaviours, barriers to accessing health care) - Psychological, cognitive, and mental well-being - Social needs - Living arrangement support - Homecare, long-term care, nursing home - Assistance with household necessities - Spiritual needs   Psychosocial support can come from family, friends, coworkers, community, needs-based services, cognitive therapy, government assistance, private assistance |
| Quality Statement 10: Interventional Management of Chronic Pain | People with chronic pain are offered interventional management, when clinically indicated, in combination with other strategies and interventions as part of an interprofessional approach to chronic pain management.  **Interventional pain management** involves diagnosing or treating pain by injecting medications and/or using radiofrequency lesioning or pulsed treatment with an implanted device at sites identified as or suspected to be the mechanism of pain or the location where the pain is felt.   - Cranial nerve blocks/deep nerve blocks of the head and neck - Neuraxial blocks (including spinal cord stimulation) - Peripheral nerve blocks - Plexus blocks - Sympathetic nerve blocks - Intravenous infusions - Joint blocks - Neuroablative blocks (radiofrequency ablation) - Injections into tissues that are superficial and easily accessible (e.g., tendon, muscle, peripheral joints) may also be offered, such as trigger point and botulinum toxin (Botox) injections.   Other strategies and interventions May include, but are not limited to, strategies and interventions discussed in other quality statements (statements 3-9). | **Percentage of people with chronic pain receiving interventional management who receive interventional management as part of an approach that includes other pain management strategies and interventions**  • Denominator: total number of people with chronic pain who receive interventional management  • Numerator: number of people in the denominator who receive interventional management as part of an approach that includes other pain management strategies and interventions | **Quality indicator 10:** % of patients receiving multimodal pain management out of those with an interventional pain management plan = (# with intervention with other strategy^a^ / # with intervention^b^) x 100  ^a^Any one other interventional strategy in assessment form as per standards 3-9  ^b^Intervention evidence in assessment form as per list in chart abstraction column |
| Quality Statement 11: Access and Referral to an Interprofessional Chronic Pain Program | **Interprofessional chronic pain program**  Interprofessional chronic pain programs use a variety of methods to reduce pain, physical disability, and emotional distress; enhance quality of life; improve function and self-management; and optimize the use of health care resources. Pain management strategies are applied flexibly and may include **pharmacotherapies, physical and psychologically based interventions, physical activity, therapeutic exercise, education, and techniques for self-management.** Shared decision making, goal setting, identifying and managing barriers to activity, and practising specific practical and psychological skills are used to produce sustainable positive changes in pain and function.  Pain programs **are time limited**. A return to primary care is coordinated to support the continuity of care. Short- and long-term outcomes are evaluated. Interprofessional pain programs can be offered in a **variety of settings, including hospitals and community-based clinics, and through telemedicine** | **Process Indicators:**  **Percentage of people with chronic pain whose quality of life is significantly impacted by their chronic pain who are referred to an interprofessional chronic pain program**  • Denominator: total number of people with chronic pain whose quality of life is significantly impacted by their chronic pain  • Numerator: number of people in the denominator who are referred to an interprofessional chronic pain program  **Percentage of people with chronic pain who have been referred to an interprofessional chronic pain program who have their first contact with the program within 3 months after referral**  • Denominator: total number of people with chronic pain who have been referred to an interprofessional chronic pain program  • Numerator: number of people who have their first contact with an interprofessional chronic pain program within 3 months after referral  **Structural Indicator:**  Local availability of interprofessional chronic pain programs | **Quality indicator 11:** % of patients referred to the chronic pain clinic who are assessed within 3 months of the referral = (# of patients assessed within 3 months / total # of people referred to chronic pain clinic) x 100 |
| Quality Statement 12: Transitions and Coordination of Care | **Collaborative care** an interprofessional, team-based approach that includes the person with chronic pain and their family or caregiver as team members, as well as other specialty consultation support, as needed. Collaborative care includes:   - Dedicated staff to coordinate, support, and educate patients - Methods for reliable and systematic patient follow-up - Consistent use of evidence-based treatment practices - Communication between providers - A detailed written plan that helps team members to understand the care expected and to monitor adherence to quality care | **Process indicators:**  **Percentage of people with chronic pain who transition from one health care provider or setting to another who have a documented collaborative care plan**  • Denominator: total number of people with chronic pain  who transition from one health care provider or setting  to another  • Numerator: number of people in the denominator who  have a documented collaborative care plan  **Percentage of people with chronic pain who have transitioned from one health care provider or setting to another who are satisfied with their transition**  • Denominator: total number of people with chronic pain who have transitioned from one health care provider or setting to another  • Numerator: number of people in the denominator who  are satisfied with their transition  **Percentage of people with chronic pain who have transitioned from one health care provider or setting to another who report that there was good communication between their health care providers**  • Denominator: total number of people with chronic pain who have transitioned from one health care provider or setting to another  • Numerator: number of people in the denominator who report that there was good communication between their health care providers | **Quality indicator 12:** % of patients with documented communication between a member of the chronic pain clinic team with referring provider = (# with communication with referring provider / total # with chronic pain) x 100  Evidence of communication with referring health care provider:   - Follow-up letter to referring provider in chart - Documented communication (phone, email, patient portals) with referring provider - Documented collaborative care plan with referring health care provider |

Data for assessing alignment of care for each quality standard with the Ontario Health Quality Standard: Chronic pain care for adults, adolescents, and children will be collected through extraction from the local electronic medical record.

**References**

1. Quality Standards: Chronic Pain Care for Adults, Adolescents, and Children. Quality Standards Ontario Health; 2019.

2. Reiman MP, Manske RC. The assessment of function: How is it measured? A clinical perspective. The Journal of manual & manipulative therapy. 2011;19(2):91-9.

3. Caspersen CJ, Powell KE, Christenson GM. Physical activity, exercise, and physical fitness: definitions and distinctions for health-related research. Public Health Rep. 1985;100(2):126-31.

4. Riebe D, Ehrman JK, Liguori G, Magal M. ACSM’s Guidelines for Exercise Testing and Prescription. Tenth ed. Liguori G, Magal M, editors. Philadelphia: Wolters Kluwer; 2018.
